# What place does nurse‐led research have in the COVID‐19 pandemic?

**DOI:** 10.1111/inr.12660

**Published:** 2021-02-10

**Authors:** E. Castro‐Sánchez, A.M. Russell, L. Dolman, M. Wells

**Affiliations:** ^1^ School of Health Sciences City, University of London London UK; ^2^ NIHR Senior Nurse Research Leader Imperial College Healthcare NHS Trust London UK; ^3^ Senior Lecturer, College of Medicine and Health University of Exeter South Cloisters St Luke’s Campus Exeter UK; ^4^ Surgery and Cancer Imperial College London London UK

**Keywords:** COVID‐19, Health Policy Research, Infection Control, Nursing Capacity Building, Nursing Policy, Nursing Research, Pandemic

## Abstract

**Aim:**

Reflect upon the visibility of nursing‐led research during the COVID‐19 pandemic.

**Background:**

The emerging SARS‐CoV‐2 infection has galvanized collaborative and multidisciplinary efforts in clinical and research practice worldwide. The scarce evidence‐base to manage patients with COVID‐19 has included limited nurse‐led research.

**Introduction:**

Clinical research nurses have greatly contributed to the delivery of COVID‐19 research, yet the number of COVID‐19 nursing‐led research papers appears to be limited, with even fewer nurse‐led research projects funded.

**Methods:**

Authors’ views and PubMed search on ‘COVID‐19 and nursing’.

**Findings:**

There is a dearth of nursing‐led research. Most papers describe the nursing contribution to COVID‐19 care, changes in nursing working arrangements and emotional burden. There are opportunities to explore the consequences to vulnerable population groups of public health measures implemented to stop the progress of the COVID‐19 pandemic.

**Discussion:**

Workforce gaps, limited integration in research structures and clinical redeployment may have hampered nurse‐led research. COVID‐19 may exacerbate staffing deficits by disrupting the education pipeline, obstructing the transition from clinical to academic practice, particularly in areas where clinical academic roles are yet to emerge.

**Conclusion:**

The absence of nurse‐led research in COVID‐19 can be explained by chronic, underlying factors and the features of the pandemic response. Emerging models of care, effective staffing and inequalities related to COVID‐19 appear obvious research areas. Nursing leadership needs to strengthen its political voice and lobbying skills to secure nurse‐led research funding.

**Implications for Nursing Policy:**

Embracing international nursing research, strengthening collaborations and lobbying policymakers for investment in nurse‐sensitive research would enhance the response to COVID‐19.

## Introduction

In just a few months, research collaborations worldwide have erupted to answer urgent questions on the viral genome, public health interventions, pharmacological therapies and potential vaccines (Folegatti et al. 2020), all in response to the COVID‐19 pandemic. In this paper, we reflect on how these alliances have galvanized an impressive clinical, academic and social effort, with stakeholders from health care, industry and charitable organizations, and lament the relative absence of nurses and nursing‐led research from the research table.

The central role of nurses in the delivery of COVID‐19 research has already been highlighted (Iles‐Smith et al. 2020; Jones et al. 2020). Without clinical research nurses, the public health response to the pandemic would have been severely compromised in the UK (National Institute for Health Research 2020) and globally (van Dorn 2020; Xiao & Jiang 2020). Institutional changes aimed at facilitating the rapid approval of studies, such as abbreviated ethical review forms, may have eased the workload of clinical research nurses. However, they have still experienced additional challenges of managing multiple research studies alongside clinical redeployments (Chen et al. 2020).

A recent editorial pointed out that responses to many of the pandemic challenges had foundations in nursing research, including telehealth interventions, safe and effective staffing, managing ethical dilemmas, stress and burnout (Lake 2020). To date, however, little COVID‐19‐related nursing research appears to have been funded or published (UK Collaborative on Development Research 2020; US National Institutes of Health 2020).

## Methods

A PubMed search on ‘COVID‐19 and nursing’ highlights that the majority of published papers reflect opinions on the impact of COVID‐19 on nursing working arrangements, the nursing contribution towards the care of patients with SARS‐CoV‐2, or the psychological and emotional burden borne by nurses caring for these patients. For example, Liu et al. (2020) described the lived experience of nurses in China during the pandemic debut, depicting situations which would resonate worldwide. Similarly, Al Thobaity & Alshammari (2020) reviewed the impact of COVID‐19 on bedside nurses, stressing gaps in human and material resources, and an emotional toll comparable to what disaster and first responders experience. It would not surprising then that, for the exhausted and scarce nurses available on the clinical frontline, engaging in research may appear as an unreachable aspiration.

These research experiences certainly make an important contribution to understanding the pandemic. But high quality, nurse‐led, interdisciplinary research is required to shape contemporary clinical practice and expand professional boundaries. Effective treatments are still few and supportive care remains the cornerstone of therapeutic management. The absence of research on nursing interventions is both surprising and disappointing, not least because 2020 was been designated the International Year of the Nurse and the Midwife by the World Health Assembly, putting our profession in the spotlight.

## Discussion

Why are nursing‐focussed studies scarce in COVID‐19 research? The underpinning reasons for an apparently slow response to funding and research opportunities are complex. The pandemic may have acted as an unforeseen stress test, exacerbating and bringing key existing deficiencies to light.

Firstly, nursing shortages remain a fundamental problem, with ~40 000 vacancies in England alone and 5.9 million globally. This deficit in human capital impacts quality of care in patients with SARS‐CoV‐2 (Padula & Davidson 2020) but also existing nursing staff (Lasater et al. 2020). The disruption to educational pipelines caused by COVID‐19, with limitations on learning environments due to pandemic mitigation measures such as lockdowns or social distancing, may discourage aspiring nursing candidates and further slow or interrupt the supply of newly‐qualified professionals. Moreover, calls for fast‐tracked and sustained investment in nursing to strengthen the global response to COVID‐19 do not seem to explicitly advocate for funding for more nurse researchers (Rosa et al. 2020), an area which requires continued and considered lobbying.

Secondly, investment in clinical academic leadership for nurses has been out of step with medical professions for some time. For example, 306 nurses currently hold substantive chairs in UK Higher Education institutions, less than 0.05% of nursing and midwifery active registrants, and clinical academics make up 0.1% of the nursing, midwifery, and allied health professional workforce, compared with ~5% in the medical profession (Council of Deans 2018). The situation across the world does not seem any more favourable, with structural hurdles such as absent formal clinical academic training pathways in Australia, scarce –yet increasing – opportunities in the US (Carter et al. 2020), or insufficient research investment and policy incentives in China (Carrick‐Sen et al. 2019), to cite some examples. The acute shortage of nurses and nursing faculty afflicting African countries would limit opportunities to expand training, education, and research capacity due to few doctoral‐educated nurses, restricting even more the prospects for nurses to enrol in doctoral programmes (Bvumbwe & Mtshali 2018; Sun et al. 2017; Sun & Larson 2015). The few clinical academic or faculty nurses may also be attracted to migrate, for professional or financial reasons (Labonté et al. 2015).

Thirdly, the redeployment of many clinical academic and research nurses enforced by the COVID‐19 response meant that research expertize in nursing was depleted at a crucial time. In addition to the impact on established clinical academics, this provisional transference from research to practice may have been particularly felt by nurses aspiring to establish early research careers, who may end up disproportionately disadvantaged if they have had to halt studies, or pause the writing of manuscripts or funding applications due to redeployment, or simply physical and emotional exhaustion to progress such outputs. Rapid modifications in the type, scale or range of research opportunities allowed or approved in order to comply with pandemic measures may also foster a glut of studies in few overcrowded fields, which may hamper the progress of early career nursing researchers.

For these researchers, travel restrictions and cancellation of conferences and exchanges further limit prospects for internationalization, global collaboration and mentorship which would normally serve to break down the traditional political, policy and professional barriers to nursing research and foster opportunities to increase its global quality (Sun et al. 2017). Without these opportunities, the exchange of information for mutual learning between clinical academic nurses in high‐ and low‐ and‐middle‐income countries would be more difficult, at a time when the dissemination of innovations developed to respond to pandemic surges is most critically needed (Catton 2020; United Nations Innovation Network 2020).

The lack of COVID‐19 nursing research outputs may also reflect existing weaknesses in the integration of nursing research and practice. Local, national and international structures do not enable prompt mobilization of nursing‐focused research teams, and an unconscious tendency to default to a ‘command and control’ model of nursing leadership, as witnessed during the COVID‐19 pandemic, does not facilitate nursing research visibility (Rosser et al. 2020). Potential approaches promoted to mitigate such fragmentation of research and practice structures include joint appointments between academic and healthcare organizations, affording greater institutional integration and individual and team collaboration, and facilitating student research supervision, particularly if such research is of mutual benefit and interest for the academic and healthcare organization (WHO 2020a).

The post‐pandemic reconstruction affords an unexpected opportunity to outline a better future for nursing research. Obvious possibilities are related to data science and staffing research. Unfortunately, the quality and quantity of routine data available for research is likely to have been affected by the swift de‐escalation in documentation and recording of observations that was widely adopted to reduce nurses’ workload, particularly in intensive care. Research‐based solutions to optimizing the safety and efficiency of nursing documentation are urgently needed and could be aided by artificial intelligence tools such as speech recognition (Goss et al. 2019).

Models of safe and effective staffing and fast deployment appear as another obvious research area, including ideal approaches to such deployment and skill‐enhancing work. Some useful examples have already reported on multimodal initiatives increasing awareness of infection prevention and control behaviours and practice, transferring skills from outpatient to inpatient settings and supporting staff mobilization from inpatient to intensive and critical care settings (Placido 2020).

Other educational studies have embraced rapid ‘design thinking’ to keep abreast of the pace imposed by the pandemic response (Thakur et al. 2020). Many other interventions reported have taken advantage of technological platforms such as simulation (Dieckmann 2020), or online and blended curricula which have facilitated interactions between nursing students and lecturers and overcome lockdowns, as seen in India (Negi & Parel 2020). This burgeoning of technology‐based pedagogies in nursing, however, may have consequences on the communication skills or compassion of students once deployed in clinical practice (Dean et al. 2020).

Research is urgently needed in fundamental areas of nursing such as respiratory care, tissue viability, infection prevention and control, and nutrition, with a particular focus on the delivery of compassionate and individualized care, which is not only at the heart of palliative and end‐of‐life care but is also the key determinant of patient experience (Fadul et al. 2020). The UK National Institute for Health Research‐funded COVID Nurse Study (ISRCTN13177364) offers hope in this area of nursing‐led research. This randomized controlled trial will test a fundamental care nursing protocol for SARS‐CoV‐2 patients ventilated non‐invasively and admitted to inpatient wards. Whilst the trial is conducted in the UK, it will enable generalization of the intervention and findings from this study to other environments such as care homes, and global health systems.

More widely, multidisciplinary research must untangle the social and ethnic gradient of infection and morbidity (Pareek et al. 2020), for patients and citizens as well as healthcare workers including nurses. This research should avoid a narrow interest on the biological aspects of the COVID‐19 pandemic, downplaying the synergy between structural determinants such as precarious employment, overcrowding, and other sociodemographic variables (Patel et al. 2020). Horton (2020) has already claimed a ‘syndemic’ perspective, pointing out that the effects of socioeconomic disparities and non‐communicable diseases which predispose to coronavirus infection must be considered within interventions to address the pandemic.

This socioeconomic lens should be applied as well to research on the unintended consequences of the lockdown on the health and wellbeing of particularly vulnerable populations. These adverse effects can be wide ranging, from increases in domestic violence (Bradbury‐Jones & Isham 2020) and gambling (Auer et al. 2020), further compounded by the reduction in available mental health services (WHO 2020b). The nursing perspective of advocacy and social justice has an important contribution to make here, expanding work on other public health threats such as racism (Booker et al. 2020) or poverty (Corburn et al. 2020), with further research on optimal nurse‐sensitive approaches to influence local, national and international policymakers in pandemic‐related decisions in view of the prominence given to expert advisory panels where nurses have been, largely, absent (Santillan‐Garcia et al. 2020).

## Implications for nursing policy

Our viewpoint is primarily anchored in our experiences of UK research and practice, but we suspect that it is translatable globally. Embracing international contexts, strengthening and fostering collaborations of nurse researchers, lobbying policymakers for investment in nurse‐sensitive research would be a fitting response to what we hope is a once‐in‐a‐generation challenge.

## Author contributions

Ideation: ECS, MW; Manuscript writing: ECS, AMR, LD, MW; Critical revisions for important intellectual content: ECS, AMR, LD, MW.
